# Bilateral hip arthroplasty: is 1-week staging the optimum strategy?

**DOI:** 10.1186/1749-799X-5-84

**Published:** 2010-11-06

**Authors:** Henry D Atkinson, Christopher A Bailey, Charles A Willis-Owen, Roger D Oakeshott

**Affiliations:** 1 Department of Trauma and Orthopaedics and North London Sports Orthopaedics, North Middlesex University Hospital, Sterling Way, London N18 1QX, UK; 2 Sportsmed SA, 32 Payneham Road, Stepney 5069, Adelaide, South Australia, Australia

## Abstract

Seventy-nine patients underwent bilateral hip arthroplasty staged either at 1 week (Group 1) or after greater intervals (as suggested by the patients, mean 44 weeks, range 16-88 weeks) (Group 2), over a five year period at one Institution. Sixty-eight patients (29 bilateral hip resurfacings and 39 total hip replacements) completed questionnaires regarding their post-operative recovery, complications and overall satisfaction with the staging of their surgery.

There was no significant age or ASA grade difference between the patient groups. Complication rates in the two groups were similar and overall satisfaction rates were 84% in Group 1 (n = 32) and 89% in Group 2 (n = 36). Cumulative hospital lengths of stay were significantly longer in Group 1 patients (11.9 days vs 9.1 days)(p < 0.01); this was true for both hip resurfacing and total hip arthroplasty patients, however resurfacing patients stays were significantly shorter in both groups (p < 0.01). Postoperative pain resolved earlier in Group 1 patients at a mean of 20.9 weeks compared with a cumulative 28.9 weeks (15.8 and 13.1 weeks) for Group 2 patients (p = 0.03).

The mean time to return to part-time work was 16.4 weeks for Group 1, and a cumulative 17.2 weeks (8.8 and 8.4 weeks) for Group 2. The time to return to full-time work was significantly shorter for Group 1 patients (21.0 weeks, compared with a cumulative 29.7 weeks for Group 2)(p < 0.05). The time to return to both full and part-time work was significantly shorter in total hip replacement patients with 1-week staging compared with delayed staging (22.0 vs 35.8 weeks (p = 0.02), and 13.8 vs 19.3 weeks (p = 0.03) respectively).

Hip resurfacing patients in Group 2 had significantly shorter durations of postoperative pain and were able to return to part-time and full time work sooner than total hip arthroplasty patients. There was a general trend towards a faster recovery and resumption of normal activities following the second operation in Group 2 patients, compared with the first operation.

Bilateral hip arthroplasty staged at a 1-week interval resulted in an earlier resolution of hip pain, and an earlier return to full-time work (particularly following total hip replacement surgery), with high levels of patient satisfaction and no increased risk in complications; however the hospital length of stay was significantly longer. The decision for the timing of staged bilateral surgery should be made in conjunction with the patient, making adjustments to accommodate their occupational needs and functional demands.

## Introduction

The optimum timing for bilateral hip arthroplasty is still under debate. Single-episode sequential bilateral hip arthroplasty though potentially financially advantageous and with shorter rehabilitation periods than staged arthroplasty [1-6], has been associated with a significantly increased risk of pulmonary complications, post-operative anaemia and heterotopic ossification [6-12].

Sequential bilateral total hip replacements during the same hospitalisation period have been advocated to avoid these potential complications whilst maintaining the functional benefits of near simultaneous surgery; and good clinical results and implant survivorship has been previously reported for these patients [6].

This study compared the post-operative recovery, complications and overall satisfaction rates of patients undergoing one-week staged bilateral hip arthroplasty surgery during the same hospitalisation period with those undergoing surgery staged at intervals as suggested by the patient.

## Patients and Methods

Patients with bilateral hip osteoarthritis were treated with bilateral hip resurfacing (HR) or total hip replacement (THR) surgery. HRs were de facto offered to all patients unless contraindicated (by an age greater than 75 years, abnormal femoral head and neck morphology, femoral neck osteopenia as confirmed with bone mineral densitometry, or a patient preference for a THR).

Hip resurfacings were performed by the senior author using the Articular Surface Replacement (ASR) (Depuy Orthopaedics, Warsaw, Indiana) uncemented acetabular and cemented femoral components. All procedures were performed under general anaesthesia using a posterior approach and the femoral component was positioned using computer navigation (ASR Ci, Depuy Orthopaedics, Warsaw, Indiana/Brainlab, Feldkirchen, Germany).Total hip replacements were performed by the senior author under general anaesthesia using an anterolateral approach. The procedure was performed using an ASR uncemented acetabular component, uncemented Summit femoral stem and ASR XL metal femoral head (Depuy Orthopaedics, Warsaw, Indiana).

Drains were placed in all patients for forty-eight hours post-operatively and intravenous antibiotics were administered until drains were removed. Wound closure was performed using a non-absorbable subcuticular suture which was removed at two weeks. Patients were mobilised on the first post-operative day and low molecular weight heparin thromboprophylaxis was administered until discharge from hospital. After discharge, aspirin 150mg daily was prescribed for six weeks.

All patients were seen pre-operatively by a consultant physician to assess their fitness for anaesthesia. Patient ASA (American College of Anaesthesiologists) grades were recorded. Patients without medical contraindications were offered one-week staged bilateral procedures during the same hospital admission (Group 1). Those patients who declined one-week staging or who had medical contraindications were allowed to choose when they wished to undergo the contralateral procedure (Group 2). Patients were not randomised to a staging regime as it was apparent that the same schedule might not suit all the patients and that the post-operative requirements might differ between patients such as those in self employment and those who were retired.

All patients were sent a questionnaire evaluating the time taken for their post-operative recovery, return to daily activities (leisure activities, sport, work), surgical complications and overall satisfaction with the timing of their surgery. Patient case-notes were also reviewed. Statistical analyses were performed using Microsoft Excel statistical software. Parametric data were analysed using an unpaired two-tailed T-test. Power analysis (alpha 5%, beta 20%) indicated a minimum sample size of twenty four patients in each group to detect a difference in the average values for time to resolution of pain.

## Results

Seventy-nine patients underwent bilateral hip arthroplasty between August 2003 and August 2008. Sixty-eight patients returned completed questionnaires; of those who did not return questionnaires six had 1-week staged operations. Of those patients included in the analyses, forty were male and twenty eight female with a mean age of 58.2 years (range 36-80 years). Twenty-nine underwent bilateral HR and thirty-nine bilateral THR surgery. There were thirty-two patients in Group 1 and thirty-six patients in Group 2 (Table 1); 8 patients had been allocated to Group 2 for medical reasons, including three Jehovah's witnesses; the remaining patients had chosen to delay the staging of their surgery for personal reasons. Eight of the thirty-two Group 1 patients were retired. Most were in full-time employment working in physically demanding occupations; including three farmers, two policemen, two carpenters, two labourers, a sports coach, an electrician, a welder, a timber packer, a fireman, and a truck driver; other professions included two teachers, two company directors, a radiologist, a nurse, an ultrasonographer, a journalist, and a salesman. Seventeen of the thirty-six patients in Group 2 were retired. Patients in this group included three farmers, three office workers, three sales representatives, two housewives, a service manager, a grazier, a secretary, a civil engineer, a labourer, a teacher, an exploration geologist, and an architect.

**Table 1:** Patient Demographics

		Number of Patients	Mean Age (Years)	Mean ASA grade	Male: Female
**Group 1**	**Hip Resurfacings**	14	51.7		10:4
					
	**Total Hip Replacements**	18	61.9		11:7
	
	**All Group 1**	32	57.4	1.91	21:11

**Group 2**	**Hip Resurfacings**	15	52.1		6:9
					
	**Total Hip Replacements**	21	63.7		13:8
	
	**All Group 2**	36	58.9	2.11	19:17

**All Patients**		68	58.2	2.01	40:28

There were no significant differences between the ages (p = 0.59) or ASA grades (p = 0.09) between the groups, though there was a trend towards a higher ASA grade in Group 2 patients (Table 1). Group 1 had a larger proportion of men, and Group 2 a larger proportion of retirees. Patients undergoing HR were significantly younger than those undergoing THR (p < 0.01) in both groups, reflecting either a greater number of contraindications to hip resurfacing or a preference for THR amongst our older patients. The mean interval between procedures in Group 2 was 44 weeks (range 16-88 weeks). Mean follow-up from the date of initial surgery was 34 months (range 12 to 60 months).

Cumulative lengths of hospital stay were significantly longer in Group 1 patients (11.9 days compared with 9.1 days for Group 2 patients)(p < 0.01) (Table 2); this was true for HR and THR patients. HR patients' hospital stays were significantly shorter than THR patients in both groups (p < 0.01) (Table 2). Group 1 HRs stayed for a mean of 11.1 days, while Group 2 HRs stayed a cumulative 7.3 days (3.6 and 3.7 days). Group 1 THRs stayed for a mean of 12.6 days, while Group 2 THRs stayed a cumulative 10.4 days (5.2 and 5.2 days).

**Table 2:** Results

	Cumulative hospital length of stay (days)	Cumulative time until pain-free (weeks)	Time to independent living (weeks)	Return to leisure activities (weeks)	Return to sport (weeks)	Return to work-Part time (weeks)	Return to Work - Full time (weeks)
**All Hip Arthroplasty**							

**Group 1**	11.9	20.9	11.7	13.4	24.5	14.0	21.0

**Group 2**	9.1; (4.5, 4.6)	28.9; (15.8,13.1)	17.4; (9.3, 8.1)	22.2; (12.6, 9.6)	32.0; (17.1, 14.9)	17.2; (8.8, 8.4)	29.7; (15.4, 14.3)

**p-value**	**p < 0.01; **(p = 0.81)	**p = 0.03; (p < 0.01)**	**p = 0.02**; (p = 0.25)	**p < 0.01; (p < 0.05)**	p = 0.21; (p = 0.50)	**p = 0.04**; (p = 0.72)	**p < 0.05**; (p = 0.65)

**Hip Resurfacing**							

**Group 1**	11.1	16.9	11.1	15.7	24.2	14.1	20.2

**Group 2**	7.3; (3.6, 3.7)	26.0;(14.5, 11.5)	15.6; (8.1, 7.5)	22.4; (12.1, 10.3)	34.0; (18.3, 15.7)	15.1; (7.5, 7.5)	22.9; (12.2, 10.7)

**p-value**	**P < 0.01; **(p = 0.59)	**p = 0.04; (p = 0.04)**	p = 0.19; (p = 0.47)	p = 0.16; (p = 0.43)	p = 0.33; (p = 0.67)	p = 0.60; (p = 1.0)	p = 0.66; (p = 0.65)

**Total Hip Replacement**							

**Group 1**	12.6	24.1	12.1	11.5	24.8	13.8	22.0

**Group 2**	10.4; (5.2, 5.2)	31.0;(16.7, 14.3)	18.7; (10.2, 8.5)	22.0; (12.9, 9.1)	30.8; (16.3, 14.4)	19.3; (10.0, 9.3)	35.8; (18.2, 17.6)

**p-value**	**p < 0.01**; (p = 1.0)	p = 0.22; **(p = 0.03)**	p = 0.06; (p = 0.34)	**p < 0.01; **(p = 0.06)	p = 0.44; (p = 0.62)	**p = 0.03**; (p = 0.67)	**p = 0.02**; (p = 0.81)

**Comparing HR and THR in Group 1**	11.9, 12.6 **p < 0.01**	16.9, 24.1 p = 0.34	11.1, 12.1 p = 0.82	15.7, 11.5 p = 0.25	24.2, 24.8 p = 0.92	18.6, 13.8 p = 0.30	20.2, 22.0 p = 0.76

**Comparing HR and THR in Group 2**	7.3, 10.4 **p < 0.01**	26.0, 31.0 **p = 0.02**	15.6, 18.7 p = 0.23	22.4, 22.0 p = 0.93	34.0, 30.8 p = 0.73	15.1, 19.3 **p < 0.05**	22.9, 35.8 **p = 0.03**

Key: HR - Hip Resurfacing, THR - Total Hip Replacement

P-values: The first figure compares Groups 1 and 2, the second figure (in parentheses) compares differences between consecutive operations in Group 2 patients.

The mean time to complete resolution of hip pain was significantly shorter in Group 1 patients (20.9 compared with a cumulative 28.9 weeks (15.8 and 13.1 weeks) for Group 2 patients (p = 0.03)(Table 2). Further analysis determined that this difference was due to a significantly shorter duration of pain in Group 1 HR patients compared with Group 2 HR patients (26.0 versus 16.9 weeks)(p = 0.04); while there was no significant difference in pain duration for THR patients between the groups (p = 0.22). Group 2 HR patients also had a significantly shorter cumulative duration of pain than did Group 2 THR patients (26.0 versus 31.0 weeks)(p = 0.02).

The mean time for returning to part-time work was 14.0 weeks for Group 1, significantly shorter than a cumulative 17.2 weeks (8.8 and 8.4 weeks) for Group 2 patients (p = 0.04). The mean time for returning to full-time work was significantly shorter for Group 1 patients (21.0 weeks compared with a cumulative 29.7 weeks for Group 2)(p < 0.05). A further analysis showed that these differences were due to a significantly shorter total time off work in Group 1 THR patients compared with Group 2 THR patients (22.0 versus 35.8 weeks)(p = 0.02). Group 1 THR patients were also able to return to part-time work significantly earlier than Group 2 THR patients (13.8 versus 19.3 weeks)(p = 0.03).Differences between Group 1 and 2 HR patients were not significant. Group 2 HR patients were able to return to part-time and full-time work, and leisure activities significantly earlier than Group 2 THR patients. Group 1 patients were able to return to independent living significantly sooner than Group 2 patients (p = 0.02), even when corrected for patient age (p < 0.05). There were no significant differences in the time taken to return to sporting activities between the groups. There was a general trend for Group 2 patients to have a faster recovery and an earlier resumption of normal activities following their second operation, compared with their first operation.

All patients were asked whether they would have surgery staged in the same way again. Twenty-seven (84%) Group 1 patients stated they would, one was not sure and four stated they would not. These four patients would have rather had their surgery staged more than six months apart; 1 of these patients was retired and 2 had heavy labouring jobs. Twenty-nine Group 2 patients (81%) stated they would have surgery staged in the same way again and seven would not. Of these seven patients, six patients would have preferred the interval between operations to be shorter (4 retirees) and one patient (teacher) would have preferred a longer interval between procedures.

Twenty-seven of the Group 1 patients (84%) were either satisfied or very satisfied with the staging of their surgery. Three patients had been neither satisfied nor dissatisfied, and two patients were very dissatisfied with the staging of their surgery. Thirty-two of the Group 2 patients (89%) were either satisfied or very satisfied, two were neither satisfied nor dissatisfied, and two patients had been dissatisfied.

Patient-reported post-operative complication data is shown in Table 3. Six patients in Group 1 and seven in Group 2 described hip pain as a complication. One Group 1 patient who had undergone bilateral staged total hip replacements had persistent pain in one hip and subsequently underwent a revision procedure twelve months postoperatively at a different hospital. There were no significant differences in wound or urinary tract infections, leg length discrepancy, abductor detachment, deep vein thrombosis or pulmonary embolus rates between the two groups. Four Group 1 patients attributed their complications to the timings of their surgery. One patient had required oral antibiotics for a superficial wound infection following hip resurfacing, which subsequently resolved. One female patient developed a urinary tract infection after catheterisation which had been required until she was fully ambulant.

**Table 3:** Complications

	Hip Pain	Superficial Wound Infection	Urine Infection	Leg Length Discrepancy	Abductor Detachment	Deep Vein Thrombosis	Pulmonary Embolus
**All Hip Arthroplasty**	n = 68						

**Group 1**	6	4	2	1	2	0	0

**Group 2**	7	4	1	3	0	1	1

**Hip Resurfacing**	n = 29						

**Group 1**	1	3	0	0	1	0	0

**Group 2**	3	1	1	0	0	1	0

**Total Hip Replacement**	n = 39						

**Group 1**	5	1	2	1	1	0	0

**Group 2**	4	3	0	3	0	0	1

## Discussion

The optimum timing for bilateral hip arthroplasty is still under debate. Single-episode sequential bilateral hip arthroplasty has been shown to have the advantages of lower costs of inpatient hospital stay and anaesthesia, a shorter overall post-operative rehabilitation time, a reduced length of time to completion of surgery and improved hip mobility due to releases of the contralateral hip contractures [1-6]. However they have been associated with a significantly increased risk of pulmonary complications, post-operative anaemia and heterotopic ossification [6-12]. Simultaneous bilateral total knee arthroplasty surgery has similarly been associated with higher rates of serious cardiac and pulmonary complications when compared with staged bilateral and unilateral total knee replacements [13].

Sequential bilateral total hip replacements during the same hospitalisation period have been advocated to avoid these potential complications whilst maintaining the functional benefits of near simultaneous surgery; and good clinical results and implant survivorship have been previously reported in these patients [6]. One-week staged bilateral total knee replacements have similarly been shown to have lower complication rates, with lower total operative blood losses than for single episode (simultaneous/sequential) or longer-interval staged procedures [14].

Cumulatively, our study showed that bilateral hip arthroplasty staged at a 1-week interval resulted in an earlier resolution of hip pain, an earlier return to independent living and leisure activities, and less cumulative time off work than surgery staged at greater intervals; this was particularly true of total hip replacement patients. The study also found that hip resurfacing patients had shorter hospital lengths of stay than total hip replacement patients with both staging regimes. Hip resurfacing patients also had a shorter duration of pain and less time off work than total hip replacement patients (in those patients having delayed bilateral hip arthroplasty).

Our study found that cumulative lengths of hospital stay were significantly longer in the 1-week staged cohort (3.8 days longer for HR and 2.2 days longer for THR patients), with resultant increased hospital costs. This was primarily due to patients being kept in hospital for a full 7 post-operative days following their first surgery; thus potentially artificially prolonging their length of stay. If one assumed that the length of stay from the first surgery was the same as that of the second surgery in Group 1 patients (with patients being sent home "on leave" between procedures), this would mean that the corrected mean cumulative lengths of stay for HR patients would be 8.2 days (twice 4.1 days), and 11.2 days (twice 5.6 days) for THR patients. The corrected values of hospital length of stay still however remain significantly longer than those of Group 2 patients (HR 8.14 days versus 7.33 days (p = 0.04), THR 11.22 versus 10.38 days (p = 0.02)). However it is likely that these increased hospital costs would be offset by savings from patients only having to undergo a single rehabilitation period; not to mention the potential cost savings of patients having a shorter overall period off-work.

Thus a one week staging regime might appeal to those patients wishing to have as little cumulative time from full-time work as possible, and the shortest overall disruption to their ability to live independently. While retired patients or those in sedentary occupations might rather prefer procedures with delayed staging, which might allow them to return (to work), leisure and sporting activities sooner (while between procedures).This rationale may explain why a higher proportion of men and those in self-employment chose one week staging on our series.

With very high levels of patient satisfaction reflected with both types of staging regime and no significant difference in observed complication rates, the decision for the timing of staged bilateral surgery should be made in conjunction with the patient, making adjustments to accommodate their occupational needs and functional demands.

Though the inclusion of different forms of hip arthroplasty and the methods of patient selection may be criticised, the numbers of hip resurfacings and total hip arthroplasties and patients demographics were broadly similar; and the rehabilitation schedules and complication rates were comparable. This study also benefitted from being a single surgeon series thus reducing the potential variability in surgical practice seen in other studies of bilateral hip staging surgery [15].

## Abbreviations

HR: hip resurfacing; THR: total hip replacement; ASA: American College of Anaesthesiologists;

## Consent

Written informed consent was obtained from all patients for their data inclusion in this and other research at our Institution. Copies of these consent forms are available for review by the Editor-in-Chief of this journal

## Competing interests

The authors declare that they have no competing interests.

## Authors' contributions

All the patients underwent arthroplasty surgery by RO. HA, CB and CWO wrote the manuscript. All authors have read and approved the final manuscript.
